# Combining Topical and Oral Botanicals for Skin Redness, Pigmentation, Sleep, and Mood: A Randomized Controlled Study

**DOI:** 10.3390/jcm11226690

**Published:** 2022-11-11

**Authors:** Jessica Maloh, Mincy Chakkalakal, Fatima Sulaiman, Waqas Burney, Cindy J. Chambers, Raja K. Sivamani

**Affiliations:** 1 Integrative Skin Science and Research, Sacramento, CA 95819, USA; 2 College of Medicine, California Northstate University, Elk Grove, CA 95757, USA; 3 Pacific Skin Institute, Sacramento, CA 95815, USA; 4 Zen Dermatology, Sacramento, CA 95819, USA; 5 Department of Dermatology, University of California Davis, Sacramento, CA 95816, USA

**Keywords:** botanicals, combined oral and topical skin care, skin redness, skin pigmentation, sleep, mood

## Abstract

External and internal stressors have been found to adversely affect skin health and overall wellness. There is growing interest in the use of anti-inflammatory and antioxidant plant-derived ingredients, such as ashwagandha, saffron, l-theanine, and tocopherol, to mitigate the impact of these stressors. In this study, we evaluate the effectiveness of oral and topical products (InnerCalm and SuperCalm, respectively) that contain naturally derived ingredients on skin redness, skin pigmentation, sleep, and mood in healthy females with Fitzpatrick skin type 1–4 and self-perceived sensitive skin. Subjects were randomized to an oral (oral group), a topical (topical group), or a combination of both the oral and topical interventions (combined group). Standardized photography-based image analysis was used to assess skin redness and pigment. Self-assessments of mood and sleep were measured with the abbreviated profile of mood states (POMS) questionnaire, and the Pittsburgh sleep-quality index (PSQI), respectively. Assessments were made at the baseline, 1-week, 4-weeks, and 8-weeks of the intervention. The average facial redness decreased in the topical group at 8-weeks (*p* < 0.001) and in the combined group at 4-weeks (*p* < 0.05) and 8-weeks (*p* < 0.001), relative to the baseline. The average facial pigmentation decreased in the oral (*p* < 0.05) and combined (*p* < 0.05) cohorts at 8-weeks, relative to the baseline. The oral group exhibited an improvement in sleep quality at 1-week relative to the baseline (*p* < 0.05) and at 8-weeks relative to the baseline (*p* < 0.05). Finally, the combined group demonstrated improvement in fatigue (*p* < 0.01) and confusion (*p* < 0.05) at 8-weeks relative to the baseline, though total mood disturbance increased in all 3 groups over the course of the study. Measured outcomes relating to mood may be confounded with the timing of the study, which ran during the COVID pandemic. Overall, we demonstrate the role of oral and topical herbs and of nutraceuticals for skin health and wellness. Further research will be needed to elucidate synergistic effects in oral and topical combination regimens.

## 1. Introduction

There are multiple external and internal contributors to skin health and appearance. These include environmental conditions, psychological factors such as stress and sleep, the use of topical formulations, and dietary intake [1,2,3,4]. To optimize treatment approaches relating to skin health, it is important to consider the interplay between skin health contributors. 

Environmental stressors, such as ultraviolet light, humidity, and heat can have adverse effects on skin health [1,5,6,7]. Ultraviolet exposure can result in the production of reactive oxygen species, the degradation of extracellular matrix components, and inflammation [8]. The consequences can include wrinkle formation, skin dryness, erythema, and hyperpigmentation [5,6,9]. Additionally, environmental conditions have been associated with self-perceived sensitive skin. One survey suggests that those with sensitive skin are significantly more likely to react to heat, cold, wind, humidity, dry weather, rough fabrics, and stress, compared to those without sensitive skin [1]. Among all these conditions, stress was most significantly associated with self-perceived sensitive skin in individuals under the age of 40 [1]. 

Animal and human studies have shown that psychological stress can adversely impact the skin [2]. The release of hormones associated with chronic stress can lead to the production of reactive oxygen species, DNA damage, and inflammation, which, in turn, can contribute to signs of aging skin [10]. Sleep dysfunction can also act as an internal stressor and affect skin. This is because insufficient sleep and/or poor sleep quality can lead to hormone and immune dysregulation, and increased production of reactive oxygen species and inflammatory markers [11]. In fact, one study found that poor sleep quality, as assessed by the Pittsburgh sleep quality index (PSQI), is associated with more water loss through the skin, decreased skin barrier recovery after disruption, decreased recovery from skin redness after UV exposure, and poorer perceptions of skin appearance [12]. In addition, relative to those with good sleep quality, poor sleep was associated with more signs of skin aging such as pigmentation and fine lines [12].

Overall, the literature suggests that both external and internal stressors impact skin health and appearance via inflammation and oxidative stress. For these reasons, optimal skin care should include anti-inflammatory and antioxidant activity against both external and internal insults.

Over the years, various topical skin-care products have been widely available in the market and have demonstrated their potential to improve skin texture, pigmentation, hydration, fine lines, and wrinkles [3]. Recently, diet and nutrition have been of considerable interest due to a rapid rise in research demonstrating their role in health and overall wellness [13,14]. More specifically, the oral and topical supplementation of herbs, botanical extracts, and various micronutrients have shown promising anti-inflammatory, antioxidant, and anti-stress properties [15]. 

However, with the increasing number of products on the market, it is important to examine the efficacy and safety of concomitant topical and oral skin-care regimens. In this randomized clinical trial, we investigated the use of topical and oral anti-inflammatory and antioxidant botanicals in the management of external and internal contributors of skin health and appearance. The oral product used, InnerCalm, contains natural ingredients, such as adaptogens, to calm the mind. The two topical products used were the SuperCalm Skin Relief Serum and the SuperCalm Soothing Hydrator, which contain ingredients to calm the skin and support barrier integrity. 

## 2. Materials and Methods

### 2.1. Study Participants, Study Design, and Intervention

This study was conducted between December 2019 and May 2020 as an 8-week randomized clinical trial approved by the Institutional Review Board by IntegReview Ltd. The study was listed on clinicaltrial.gov (NCT04872946, accessed 9 November 2022). All participants provided written informed consent prior to participation in the study. Subjects living in the Sacramento and Davis regions were recruited. The inclusion criteria consisted of females ages 18–55 years old, with Fitzpatrick skin type 1–4 and self-perceived sensitive skin. All study visits occurred at Integrative Skin Science and Research. Seventy-five women met the inclusion criteria and were randomly assigned into the three groups. 

The “oral” group received an oral supplement (InnerCalm, Arbonne^®^, Irvine, CA USA) for daily consumption, the “topical” group received a topical regimen only (SuperCalm Skin Relief Serum and SuperCalm Soothing Hydrator with Tigergrass blend, Arbonne^®^, Irvine, CA USA), and the “combined” group received a topical regimen (SuperCalm Skin Relief Serum and SuperCalm Soothing Hydrator with Tigergrass blend, Arbonne^®^, Irvine, CA, USA) for application to the face and neck daily in addition to the oral supplement (InnerCalm, Arbonne^®^, Irvine, CA, USA). 

InnerCalm’s key ingredients include l-theanine, ashwagandha, and saffron. The key ingredients in the SuperCalm Serum and Hydrator include a probiotic lysate (*Lactococcus* ferment lysate), and the herbs *Agastache mexicana* and Tiger Grass, also known as Gotu Kola or *Centella asiatica*.

Individuals with a known allergy to the study agents were not included in the study. Subjects were asked to discontinue the use of other nutritional supplements, including antioxidants, herbs, or protein-based supplements, two weeks prior to the baseline visit and throughout the study. Those who were unwilling to discontinue these supplements were excluded. The use of isotretinoin in the last 6 months was also part of the study’s exclusion criteria. The use of salicylic acid; beta hydroxy acids; topical retinoids; topical depigmenting agents; or vitamins A, C, or E was not permitted 14 days prior to the baseline visit and throughout the study. Current smokers or individuals who have smoked within the past three years were excluded from the study. Individuals who had surgical or cosmetic procedures on the face and neck in the three months prior to enrolling in the study were also excluded. Additionally, those with a diagnosis of acne, pregnant or breastfeeding women, adults unable to consent, minors, and prisoners were excluded from the study. 

### 2.2. Facial Imaging and Subjective Assessments of Mood and Sleep

Facial redness and pigmentation were assessed using high-resolution facial photographs captured and analyzed by the BTBP 3D Clarity Pro^®^ Facial Modeling and Analysis System (Brigh-Tex BioPhotonics, San Jose, CA, USA). Images were collected after subjects had adjusted to ambient conditions for fifteen minutes in a climate-controlled room. Self-assessments of sleep quality with the use of the Pittsburgh sleep quality index Survey (PSQI survey) and self-assessments of mood with the use of profiles of mood state (POMS) were collected. These parameters were assessed at pretreatment baseline, 1-week, 4-week, and 8-week time intervals. 

### 2.3. Statistical Analysis

All results are presented as mean ± standard deviation. Statistical analysis was performed using Student’s *t*-test with Bonferroni correction. A Wilcoxon matched-pairs signed-rank test was used to analyze non-parametric data. Values of (*p* < 0.05) were considered statistically significant. Each subject served as their own control as values reported at 1 week, 4 weeks, and 8 weeks were compared to the baseline values, or 1. All data were analyzed, including subjects that were enrolled in the study and those who had received any study intervention. 

## 3. Results

### 3.1. Facial Redness

**Figure 1:**
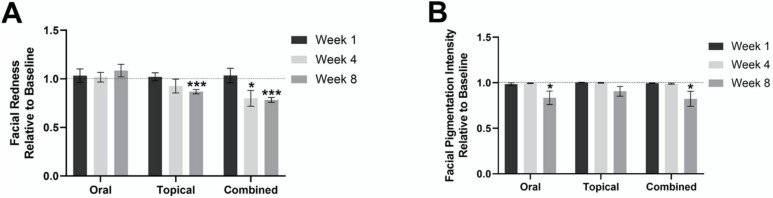
Mean facial redness and pigmentation in the oral, combined and topical groups at 1 week, 4 weeks, and 8 weeks, relative to the baseline. (**A**) Mean facial redness in the oral, combined and topical groups at 1 week, 4 weeks, and 8 weeks, relative to the baseline. (**B**) Mean facial pigmentation in oral, combined, and topical at 1 week, 4 weeks, and 8 weeks, relative to the baseline. * *p* < 0.05; and *** *p <* 0.001.

There was no statistically significant change in facial redness after 1 week, 4 weeks, or 8 weeks of oral supplementation, compared to the baseline in the oral group. A significant decrease in facial redness was observed in the combined group, a 20% ± 8.1 decrease was observed at 4 weeks relative to the baseline (*p* < 0.05), and a 22% ± 2.5 decrease was observed at 8 weeks relative to the baseline (*p* < 0.001). In the topical group, there was a significant decrease in facial redness, 13% ± 2.3, after 8 weeks of intervention compared to the baseline (*p* < 0.001). These findings are demonstrated in Figure 1A.

### 3.2. Facial Pigmentation

In the oral group, there was a statistically significant decrease, 17% ± 7.3, in facial pigmentation after 8 weeks (*p* < 0.05). A statistically significant decrease in facial pigmentation of 18% ± 8.2 (*p* < 0.05) was also observed in the combined group after 8 weeks of intervention, compared to the baseline. However, there was no statistically significant difference in facial pigmentation in the topical group at 1 week, 4 weeks, and 8 weeks, relative to the baseline. These findings are demonstrated in Figure 1B. 

### 3.3. Sleep Quality

**Figure 2:**
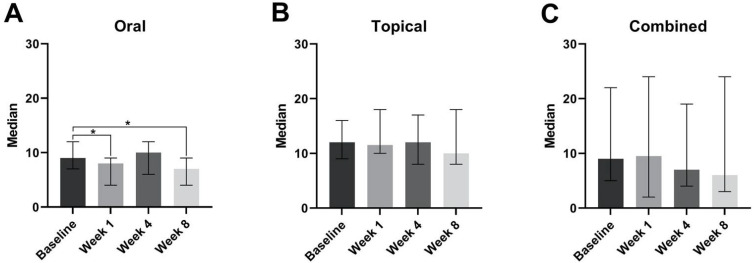
Median PSQI scores at the baseline, week 1, week 4, and week 8, with the oral (**A**), topical (**B**), or combined groups (**C**). * *p* < 0.05.

Subjects in the oral group experienced an improvement in sleep quality. This is demonstrated in Figure 2A by the significant reduction in the median PSQI scores at week 1 relative to the baseline (*p* < 0.05), and at week 8 relative to the baseline (*p* < 0.05). Those in the topical and combined groups did not experience any significant changes in median PSQI scores (Figure 2B,C).

### 3.4. Profile of Mood States (POMS)

**Figure 3:**
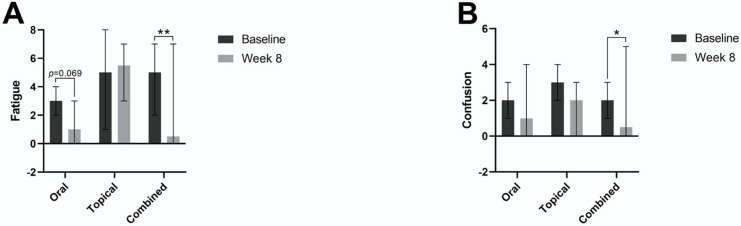
Median fatigue POMS scores (**A**) and median confusion POMS scores (**B**) with the oral, topical, and combined groups at the baseline and week 8. * *p* < 0.05; ** *p* < 0.01.

When examining individual components of mood, the combined group demonstrated significant improvement in median fatigue, from a score of 5 at the baseline to a score of 0.5 at 8 weeks (Figure 3A). We also noted an improvement in median fatigue scores in the oral group from a median score of 3 at the baseline to a median score of 1 at 8 weeks, but without significance. Improvements in fatigue were not observed in the topical group.

All three groups were also found to have a decrease in median confusion scores from the baseline to 8 weeks, but statistical significance was only found in the combined group (Figure 3B). The only other improvement noted in the POMS analysis was a decrease in tension scores in the topical group, from a median score of 3 at the baseline to a median score of 2 at 8 weeks, but this change was not found to reach statistical significance.

With regards to the overall mood states over time, there are higher total mood disturbance (TMD) scores in each of the oral, combined, and topical groups, which indicates worsening overall mood over the course of the study.

## 4. Discussion

The topical regimen given to the topical and combined groups contains *Centella asiatica* leaf extract, commonly known as Gotu Kola from Ayurveda and Traditional Chinese medicine. *C. asiatica* has been used in traditional medicine to treat various health conditions for centuries due to its diverse phytochemical profile and is currently used in the dermatological management of burns, hypertrophic scars, and eczema [16,17,18]. In our study, we report a statistically significant reduction in the appearance of facial redness in the topical group after 8 weeks (*p* ≤ 0.001). Since many studies have demonstrated the protective effect of topical antioxidant supplementation on erythema, our findings may be associated with the free radical scavenging activity of the saponins, flavonoids, tannins, and carotenoids that constitute *C. asiatica* [19,20]. Furthermore, a 2014 study also reported a statistically significant decrease in erythema through the prophylactic topical supplementation of a skin-care product containing 5% of *C. asiatica* extract for four weeks [19]. In this study, the onset of skin erythema and irritation was partially attributed to the activation of cyclooxygenase and the release of nitric oxide and proinflammatory cytokines [19,21]. The saponins of *C. asiatica* may be partially responsible for the observed results as previous studies have reported its potential to inhibit the expression of nitrogen oxide synthase and of cyclooxygenase and to prevent the release of pro-inflammatory markers and nitric oxide in the inflammatory response of the NFkB pathway [22].

Moreover, the topical regimen we studied is also formulated with *Agastache mexicana* flower/leaf/stem extract and tocopherol, which are both known for their anti-inflammatory properties [23,24]. Additionally, tocopherol is known for its ability to directly scavenge reactive oxygen species and was successfully used to reduce acute skin inflammation in a topical formulation [25]. Therefore, the anti-inflammatory and antioxidant activity of *C. asiatica leaf extract, A. mexicana* flower/leaf/stem extract, and tocopherol may be responsible for the statistically significant reduction in skin redness in the topical group after 8 weeks of product application.

Interestingly, there was an observed 9.0% ± 5.4 decrease in facial pigmentation at 8 weeks relative to the baseline in the topical group, but these results were not statistically significant. Since the topical regimen in our study is formulated with azelaic acid and tocopherol, which are currently used in the dermatological management of hyperpigmentation, we speculate that there is potential for improvement in facial pigmentation with an intervention lasting longer than 8 weeks [26,27], but clinical validation will require a longer-term study.

Of note, the combined group appeared to have a synergistic effect rather than either of the topical or oral regimens alone for modulating facial redness. For example, we report a statistically significant decrease in facial redness at both 4 weeks (*p* < 0.05) and 8 weeks (*p* ≤ 0.001) of the combined group. The oral group experienced no significant change in this parameter, and the topical group had a significant reduction only at week 8. This suggests that using the oral and topical products together may allow for a reduction in redness and for that reduction to occur earlier, compared to only using the topical or oral product alone. This may be attributed to the combined antioxidant and anti-inflammatory activities of the topical ingredients such as *C. asiatica*, *A. mexicana,* and tocopherol, which are in the SuperCalm products and ashwagandha, l-theanine, and saffron, which are in the InnerCalm supplement [28,29,30].

We also report a statistically significant decrease in skin pigmentation (*p* < 0.05) at 8 weeks relative to the baseline in the combined group and a significant change in the oral group. These results may be partially due to the depigmenting activity of ashwagandha extracts [31]. Additionally, the improvement of pigment may be related to the sleep-enhancing effects of the oral supplement, as poor sleep quality has been associated with increased skin pigmentation [12]. Overall, our study serves as a pilot study to depict the potential for synergistic actions of combining oral with topical regimens. Future studies with a longer duration and larger sample sizes will be necessary to further assess whether a combined approach leads to significantly greater reductions in skin pigment relative to an oral or topical product used alone.

It is important to note that other ingredients in the topical products of our study, such as lactococcus ferment lysate, *C. asiatica*, and sunflower oil, may offer benefits other than those described in the discussion above. To illustrate this point, lactococcus ferment lysate, a probiotic ingredient, has recently been incorporated in skin-care products, specifically due to its potential to improve the integrity of the skin barrier [32]. Furthermore, the saponins of *C. asiatica* have demonstrated collagen-stimulating activity in multiple in-vitro and in-vivo studies, which is of clinical importance in preventing skin injury and tolerating external insults [17,18,21]. Next, sunflower oil has been found to support the skin barrier in those with an inflammatory skin condition without causing erythema [33]. Overall, these ingredients may work together to maintain the structural barrier of the skin; prevent inflammatory events; and, in turn, reduce the instances of skin concerns such as redness and pigmentation that we see in this study.

Overall, we attribute the significant reduction in median PSQI experienced in the oral group to the sleep-supporting effects of the ashwagandha, l-theanine, and saffron in the InnerCalm supplement. It is interesting to note that the addition of the topical product to the oral product did not result in the attainment of statistical significance with regards to sleep improvement. It is possible that the topical product somehow blunted the effects of the oral product, but the potential mechanism at play is not well understood and may be related to the limitations of oral vs. topical use.

One of the main ingredients of InnerCalm is the adaptogenic herb, *Withania somnifera*, commonly known as Ashwagandha, which is widely used in Ayurveda and in herbal medicine. Adaptogenic herbs are thought to help an organism adapt to stressors and to offset the potential physiological consequences and ailments associated with stress [34]. There are several studies demonstrating their safety and efficacy in conditions of chronic stress, insomnia, and anxiety [35]. One randomized, double-blind, placebo-controlled trial investigated sleep quality and the use of 120 mg of ashwagandha extract taken once daily for 6 weeks [36]. Here, there was improvement in sleep relative to the baseline and relative to the placebo, as demonstrated by significant increases in total sleep time and sleep efficiency, and significant decreases in onset of sleep latency [36]. With the InnerCalm oral supplement in our study, subjects received a daily dose of 125 mg of ashwagandha.

The InnerCalm supplement also contains 200 mg of l-theanine, which is an amino acid derived from green tea, and like ashwagandha it has anti-inflammatory and antioxidant properties [28,37,38]. This amino acid has been found to be helpful for stress, anxiety, and sleep [28,37,38]. For example, a randomized controlled trial found that 200 mg of l-theanine taken daily for 4 weeks significantly improved scores in a depression scale, an anxiety scale, and the PSQI [38].

Finally, another natural supplement included in InnerCalm is saffron at 28 mg. Saffron has been found to reduce depression scores, improve social relationship scores, and support sleep [39,40]. In those with self-reported poor sleep, 14 mg of saffron taken twice daily was found to reduce insomnia severity and improve sleep quality and restorative sleep relative to placebo [40]. These sleep benefits may be associated with saffron’s anti-inflammatory, serotonergic, and GABA–ergic actions [40].

Due to the previously demonstrated benefits of ashwagandha, l-theanine, and saffron on anxiety, depression, stress, and sleep, we would expect an improvement in the TMD scores of the POMS questionnaire. While there were significant improvements in the specific aspects of mood, we did not note any improvements in the overall TMD scores. Here, it is important to note that this study was conducted during the COVID-19 pandemic, which has been associated with more prevalent symptoms of anxiety, depression, post-traumatic stress disorder, stress, and distress in a recent systematic review [41]. The investigation of these oral, topical, and combined products without the influence of an ongoing pandemic is needed to understand the impact on mood related outcomes.

This study had several limitations. Our study population was limited by a small sample size of 75 participants and was limited to women who graded themselves as having sensitive skin. Additionally, the study duration was limited to 8 weeks, supporting the need for a follow up study for a longer duration. This study did not involve a placebo group, although the comparative groups did allow for differentiation between the effects of the oral and the topical. Future studies should include an expanded population with a larger sample size that includes all skin types, men, and those that do not report sensitive skin. It is also important to note that during the time of the study, the COVID pandemic limited follow up at the later time points and was likely a confounder with the mood-related assessments. Nevertheless, we utilized a repeated-measures model where each subject served as their own control, increasing the internal validity of the measures and the measured changes.

## 5. Conclusions

In conclusion, oral and topical skin-care products with antioxidant and anti-inflammatory herbs and nutraceuticals may be clinically useful in offsetting the internal and external factors affecting skin health and wellness. Specifically, our data have demonstrated the potential for reductions in the appearance of facial skin redness and pigmentation with oral and topical regimens. Further studies are needed to further explore the potential synergistic effects of a combination regimen.
